# Supplementary material for: Balancing tourism development and habitat conservation in fragile ecosystems: A case study of the Qinghai-Tibet Plateau
Source: PLoS One. 2025 Jul 18;20(7):e0327803. doi: 10.1371/journal.pone.0327803 (PMC12273996; doi:10.1371/journal.pone.0327803)
Supplement: S2 Table — (DOCX) [file pone.0327803.s002.docx]

**S1 Table. Result types of two-factor interaction**

| **Basis of judgment** | **Interaction type** |
| --- | --- |
| q(X1∩X2)＜Min[q(X1),q(X2)] | Non-linearity attenuation |
| Min[q(X1),q(X2)]＜q(X1∩X2)＜MaX[q(X1),q(X2)] | The single-factor nonlinearity decreases |
| q(X1∩X2)＞MaX[q(X1),q(X2)] | Two-factor enhancement |
| q(X1∩X2)=q(X1)＋q(X2) | independent |
| q(X1∩X2)＞q(X1)＋q(X2) | Nonlinear enhancement |
